# Small Bowel Tumors: Clinical Presentation, Prognosis, and Outcome in 33 Patients in a Tertiary Care Center

**DOI:** 10.1155/2008/212067

**Published:** 2008-12-02

**Authors:** Mirna H. Farhat, Ali I. Shamseddine, Kassem A. Barada

**Affiliations:** ^1^Division of Hematology and Oncology, Department of Internal Medicine, American University of Beirut, Riad El-Solh, Beirut 1107 2020, Lebanon; ^2^Division of Gastroenterology, Department of Internal Medicine, American University of Beirut, Riad El-Solh, Beirut 1107 2020, Lebanon

## Abstract

*Introduction*. Small bowel cancers are rare. Accumulation of data regarding their clinical presentation, pathologic features, prognostic factors, treatment modalities, and outcome is difficult. *Methods*. This is a retrospective study of the medical records of 33 patients with small bowel cancers treated at the American University of Beirut-Medical Center over a 20-year period. *Results*. The study included 25 males (76%) and 8 females (24%). Median age at presentation was 56 years. Most common symptoms were abdominal pain (66.7%) and weight loss (57.6%). Thirteen patients presented with abdominal emergencies (39.3%). Lymphoma was the most common malignant tumor (36.4%), followed by adenocarcinoma (33.3%), leiomyosarcoma (15.2%), gastrointestinal stromal tumors (12.1%), and neuroendocrine tumors (3.0%). Tumors were located in the duodenum in 30% of patients, jejunum in 33%, and ileum in 36%. Resectability rate was 72.7% and curative R0 resection was achieved in 54.1% (13/24) of patients. 5-year survival of the 33 patients was 24.2%. *Conclusion*. Small bowel cancers are difficult to diagnose because of the nonspecific symptoms. Most patients present with advanced disease and have poor prognosis. Adenocarcinoma and duodenal location have the worst 5-year survival in contrast to stromal tumors and those with ileal location which have the best survival.

## 1. Introduction

Small bowel cancers are uncommon tumors of the gastrointestinal tract. As a result of their infrequency and variety, the accumulation of data regarding their clinical presentation, pathologic features, prognostic factors, treatment modalities, and outcome has been difficult.

This study was conducted to review the experience with small bowel tumors of 33 patients seen at the American University of Beirut Medical Center.

## 2. Patients and Methods

During a 20-year period (1986–2006), 33 patients were treated for small bowel tumors at the American University of Beirut Medical Center. Approval was obtained from the Institutional Research Board. Data obtained from the medical records were gathered regarding standard demographic data, presenting signs and symptoms, comorbid medical illnesses, diagnostic workup, operative procedures, pathologic diagnosis, and subsequent follow-up visits to document the recurrence of previous or subsequent associated primary cancers. Recurrence was defined as biopsy-proven tumor or radiologic evidence of obvious local or distant recurrence.

Periampullary tumors, secondary, and benign tumors of the small bowel were excluded. Staging of patients was done according to the TNM classification system and AJCC for small bowel tumors [1], except for lymphomas.

Survival duration was measured from the time of diagnosis to the last follow-up evaluation or death. Follow-up data was obtained from patient medical records or from telephone interviews with the patient or family members. Follow-up data was available on 30 out of 33 patients.

All data was coded and entered using “SPSS 15.0” computer program.

## 3. Results

### 3.1. Demographic Data

This study included 33 patients with histologically confirmed small bowel malignancies. They were 25 males (76%) and 8 females (24%) patients. The male to female ratio was 3.1:1 with a median age at presentation of 56 years (range: 23–77). Two-thirds of the patients were older than 50 years (Table 1).

There was a history of Crohn's disease in one patient, ulcerative colitis in another one, and celiac disease in a third one. One-third of the patients were smokers (11/33). Three patients (9.1%) had a history of another primary malignancy (mucinous cystadenocarcinoma of ovary, biopsy-proven cholangiocarcinoma, and leiomyosarcoma of the thigh).

Most complaints were nonspecific and these included in decreasing order abdominal pain (66.7%), weight loss (57.6%), followed by nausea and vomiting (27%) (see Table 1). Thirteen patients presented with emergencies such as bowel obstruction (*n* = 8, 24%) and bowel perforation (*n* = 4, 12%). Of the four patients that had perforation, three had lymphomas and one had leiomyosarcoma. Acute abdomen was the initial presentation in four patients (12%).

Endoscopy was performed on 10 out of 33 patients. It detected the tumor in 7 only (6 duodenum, 1 ileum) and it failed to detect the disease in 3 patients, all of which had ileal disease. Upper GI series was performed on 12 out of 33 patients. The disease was detected in 11 patients (4 jejunum, 6 duodenum, 1 ileum) and missed in one patient having a tumor in the ileum.

Computed tomography was used in 13/33 patients and emergent laparatomy in 4/33.

### 3.2. Characteristics of the Tumors

Four different histologic types of tumors were identified. Lymphoma was the most common tumor (36.4%), followed by adenocarcinoma (33.3%), leiomyosarcoma (15.2%), gastrointestinal stromal tumors (GISTs) (12.1%), or neuroendocrine tumors (3.0%) (see Table 2).

Table 2 shows the anatomical distribution of the tumors with respect to histology. The tumor was located in the duodenum in 30% of patients, jejunum in 33%, and ileum in 36%. Most adenocarcinomas were located in the duodenum (7/10), in contrast to most lymphomas (10/12) and all leiomyosarcomas (5/5) that were located in the jejunum and ileum.

### 3.3. Treatment

Surgical resection was carried out in 24 out of 33 patients. Resectability rate was 72.7% and R0 resection was achieved in 54.1% (13/24) of patients (see Table 3). Two patients underwent palliative double bypass surgery. Four patients were deemed unresectable because of peritoneal metastasis, locally advanced disease, and vascular invasion. The surgical procedures performed are listed in Table 4. There were four cases of tumor recurrence.

Reoperation was performed in 5 patients for the following reasons: tumor recurrence, metastasis, and postoperative complications. Emergent laparatomy was performed in 4 patients (3 lymphoma and 1 leiomyosarcoma). Chemotherapy was administered to 13 patients (39.3%).

### 3.4. Tumor Characteristics

Tumor size was available on 27 patients. Sixteen patients (59%) had tumors greater than 5 cm, 8 patients (30%) had tumors less than 3 cm, and 3 patients (11%) had tumor sizes between 3 and 5 cm.

Tumor grade was available for 12 patients (36.4%). Among the 12 patients, 8 patients (66.7%) had well or moderately differentiated tumors and 4 had poorly differentiated tumors (33.3%) (see Table 5).

TNM staging was available on 26 patients (78.7%). According to AJCC staging of small bowel malignancies (Table 5), tumors in our study were classified as stage I (*n* = 2), stage II (*n* = 9), stage III (*n* = 8), stage IV (*n* = 7) (see Table 6). Almost half of the patients presented with metastatic disease (stage III/IV). Lymph nodes (10/33) were the most common site involved by metastasis followed by the liver (5/33), lung (3/33), brain (1/33), bone (1/33), and omentum (1/33).

### 3.5. Survival

Survival analysis of the 33 patients showed that 51.5%, 33.3%, and 24.2% were alive at 1, 3, and 5 years, respectively. Three patients were lost to follow-up. Mean survival rates for the different malignancies were 22.03 months for adenocarcinomas, 52.85 months for lymphomas, 47.6 months for leiomyosarcoma, and 64.9 months for GIST.

1-, 3-, and 5-year survivals for the different tumor locations and histologies are listed in Tables 7 and 8, respectively. However, the number of patients in some categories was small which could have affected the significance of some of these rates.

## 4. Discussion

Neoplasms of the small intestine are unusual and constitute less than 3% of all gastrointestinal tract cancers [2–4]. These tumors are known for their rarity, variability, nonspecific symptomatology, diagnostic difficulties, delayed presentation, and overall poor prognosis [2].

Over 90% of cases occur in people over the age of 40 [5] with a median age at diagnosis of 55 years [6, 7] and men tend to be more affected by the tumors than women [5, 8], which is in agreement with our results.

Abdominal pain, weight loss, nausea, and vomiting were the most common presenting symptoms for small bowel tumors in this study, which is similar to findings reported in previous studies [7, 9]. Bleeding is more common with carcinomas; perforation is more common with lymphomas, while obstruction is common to all enteric malignancies [10]. No distinct presentation by pathology was evident in our study.

The lack of symptom specificity in patients suffering from small intestinal malignancies and failure to evaluate the small bowel, which is a blind spot to routine endoscopic and radiological diagnostic tests, have been considered as a contributing factor to the late presentations of the disease and eventually the delay in diagnosis [11].

Histologically, small bowel tumors are of different subtypes, with adenocarcinoma being the most frequent (47%) followed by carcinoid tumors (28%), GI lymphomas (12%), and GI sarcomas (12%) [2, 3, 7, 12]. In this study, lymphomas were the most common type, followed by adenocarcinomas, sarcomas, and stromal tumors.

On average, adenocarcinomas are distributed more proximally in the small intestine, most frequently in the duodenum followed by the jejunum and ileum, whereas lymphomas are more common distally, with nearly equal distribution between the jejunum and the ileum [5, 7, 8, 13]. Carcinoid tumors are distributed throughout the small intestines, but are located preponderantly in the ileum. Sarcomas are found most often in the jejunum [7]. In our study, adenocarcinomas were mostly located in the duodenum, while sarcomas and lymphomas were mostly located in the more distal parts of the small bowel.

Small bowel tumors were noticed to be associated with an increased incidence of second malignancies [2, 4, 13]. Treadwell and White reported second tumor occurrence in 43% of patients with primary small bowel tumors [4], whereas Ripley and Weinerman noted an 8-fold increase in second malignancies with small bowel carcinoma with 73% of the tumors occurring before the diagnosis of the small bowel malignancy [13]. The incidence of a second malignancy approached 9% in our series.

Surgical resection remains the cornerstone of therapy for these malignancies [11]. Cunningham et al. showed a significantly longer median survival for the resected group (26 months versus 11 months) [14]. Much of the information on the effectiveness of surgery for early GI lymphoma is based on limited, retrospective reviews that do not specifically compare primary surgical therapy with primary medical management are either chemotherapy, radiotherapy, or radiochemotherapy.

Regardless of surgical resection, the overall 5-year survival rate for small bowel cancers ranges between 20 and 50% [2, 6, 7, 14, 15]. Their disappointing course seems to be related to the delay in diagnosis because of the nonspecific symptoms [8]. In our study, the 5-year survival rates of GIST (50%) were higher than those of adenocarcinomas and lymphomas and are close to the 67% 5-year survival reported by Egberts et al. [16]. Therefore, early surgical intervention with a high index of suspicion is required to improve survival [17].

Given the low prevalence of this disease, few clinical trials of chemotherapy have been conducted and despite a variety of chemotherapeutic agents used to treat adenocarcinoma of the small bowel, no standard chemotherapy regimen exists for this disease. Many oncologists extrapolate information from trials of large bowel carcinomas or upper gastrointestinal tumors, and apply similar chemotherapy regimens in the treatment of advanced small bowel adenocarcinoma. In one of the largest retrospective reviews conducted, the M. D. Anderson Cancer Center (Tex, USA) reviewed 217 patients with small bowel adenocarcinoma and showed that the use of adjuvant chemotherapy administered to 59 patients had no survival benefit [6].

The most important limitation in our study is the small number of patients. This has limited our ability to determine the significance of the difference in survival among the various groups.

In summary, small bowel tumors are uncommon. They are difficult to diagnose because of the nonspecific symptoms. They also have a poor prognosis because most patients present with advanced disease. In our report, adenocarcinoma and duodenal location showed the worst 5-year survival, while stromal tumors and ileal location had the best 5-year survival.

## Figures and Tables

**Table 1:** Patient characteristics and clinical presentation.

	No. of patients	(%)
Age
* * <50	11	(33.3)
* * ≥50	22	(66.7)
Gender
* *Male	25	(75.8)
* *Female	8	(24.2)
Symptom
* *Abdominal pain	22	(66.7)
* *Weight loss	19	(57.6)
* *Nausea/vomiting	9	(27.0)
* *Bowel obstruction	8	(24.0)
* *GI bleeding	8	(24.0)
* *Anemia	7	(21.0)
* *Bowel perforation	4	(12.0)
* *Acute abdomen	4	(12.0)
* *Jaundice	4	(12.0)
* *Constipation	3	(9.0)

**Table 2:** Distribution of small bowel tumors by pathology and anatomical distribution.

	Duodenum	Jejunum	Ileum	No. (%)
Lymphoma	2	4	6	12 (36.4)
Adenocarcinoma	7	1	3	11 (33)
Leiomyosarcoma	0	3	2	5 (15.2)
GIST*	1	3	0	4 (12.1)
Neuroendocrine	0	0	1	1 (3.0)
Total(%)	10	11	12	33

**Table 3:** Treatment modalities offered to 33 patients with small bowel tumors.

Modality	No.	(%)
No treatment	4	12.1
Complete resection	12	36.3
Complete resection + chemotherapy	6	18.1
Incomplete resection	4	12.1
Incomplete resection + chemotherapy	2	6.0
Chemotherapy (palliative)	5	15.1

**Table 4:** Types of surgeries performed for small intestine tumors.

Surgery	No. of patients (26)	(%)
Whipple	5	(16.6)
Duodenal resection	2	(6.6)
Jejunal resection	9	(30)
Ileal resection	5	(16.6)
Right hemicolectomy	3	(10)
Bypass	2	(6.6)

**Table 5:** TNM staging of small bowel malignancies, excluding lymphoma. (Adapted from the AJCC Cancer Staging Manual, 6th Edition (2002), Springer New York).

Stage	Primary tumor^1^	Regional lymph nodes^2^	Distant metastasis^3^
I	T1/T2	N0	M0
II	T3/T4	N0	M0
III	Any T	N1	M0
IV	Any T	Any N	M1

^1^Primary tumor T1: tumor invades lamina propria or submucosa; T2: tumor invades muscularis propria; T3: tumor invades through muscularis propria into the subserosa; T4: tumor perforates the vascular peritoneum or directly invades other organs, including mesentery, abdominal wall, and pancreas. ^2^Regional lymph nodes N0: no regional lymph nodes metastasis; N1: regional lymph nodes metastasis. ^3^Distant metastasis M0: no distant metastasis; M1: distant metastasis.

**Table 6:** Stage and grade of small bowel tumors.

Grade	N	(%)
Well	3	(9)
Moderate	5	(15)
Poor	4	(12)
N/A	21	(64)

Stage

I	2	(6)
II	9	(27)
III	8	(24)
IV	7	(21)
N/A	7	(21)

**Table 7:** Survival of small intestine tumors at 1, 3, and 5 years by location.

	1 year (%)	3 years (%)	5 years (%)
Duodenum	50%	40%	10%
Jejunum	50%	20%	20%
Ileum	62.5%	37.5%	**37.5**%

**Table 8:** Survival of small intestine tumors at 1, 3, and 5 years by histology.

	1 year (%)	3 years (%)	5 years (%)
Adenocarcinoma	30%	20%	10%
Lymphoma	36.4%	27.3%	18.2%
Leiomyosarcoma	60%	20%	20%
GIST	100%	75%	**50**%
